# Antimicrobial structure activity relationship of five anthraquinones of emodine type isolated from *Vismia laurentii*

**DOI:** 10.1186/s12866-017-0954-1

**Published:** 2017-02-22

**Authors:** Gislaine Aurelie Kemegne, Pierre Mkounga, Jean Justin Essia Ngang, Sylvain Leroy Sado Kamdem, Augustin Ephrem Nkengfack

**Affiliations:** 1 Department of Microbiology, Faculty of Science, University of Yaoundé I, P.O. Box 812, Yaoundé, Cameroon; 2 Department of Organic Chemistry, Faculty of Science, University of Yaoundé I, P.O. Box 812, Yaoundé, Cameroon

**Keywords:** Anthraquinones, Emodine, Antimicrobial activity, Physicochemical property, Structure-activity relationship

## Abstract

**Background:**

Antimicrobial activity of anthraquinone compounds of emodine type has been reported by many authors. These compounds are found in *Vismia laurentii* (Clusiaceae), a plant used in traditional pharmacopoeia for treatment of microbial infections among others affections. The continuous identification of new compounds has raised the problem of the relation between the structure and antimicrobial properties.

**Results:**

The yeast growth kinetics parameters were not influenced by the pH variation as it was the case for the other tested bacteria. Fungicidal activities were noted for all molecules while only few of them had bactericidal activities, mostly on Gram positive bacteria. Mathematical model establishing a quantitative relationship between physicochemical properties of molecules and their fungicidal activities were obtained for *Candida albicans* and showed that physicochemical properties impacting on antifungal activity were polarizability, partition coefficient, molecular weight and hydrogen bond acceptor.

**Conclusions:**

This work demonstrated that the presence of a long aliphatic chain methoxy group substituted in position two of the emodine structure increased the antibacterial properties of the studied compounds. Moreover this antimicrobial property depends on the pH of the environment, and specifically on the polarizability and number of hydrogen bond acceptors of the compound.

## Background

Plants belonging to *Vismia* genus have been studied since 1979 [1] because of their biological activity due to secondary metabolites that they contain. Different parts of *Vismia laurentii* are used in the traditional pharmacopoeia in the treatment of different affections including microbial infections [2]. Previous chemical assessments carried out on this plant have resulted in the isolation and characterization of a great number of secondary metabolites, the most significant belonging to the xanthone, benzophenone and quinone classes. Quinones especially anthraquinones, present numerous biological activities such as antiprotozoa [1, 3], antituberculous, fungicidal [4], antioxidant [5], cytotoxic and antitumor activities [6]. Xanthones and anthraquinones are known to bind irreversibly with nucleophilic amino acids in proteins, often leading to the inactivation of proteins and loss of function [7]. The rarity of plant diseases in *V. laurentii* is explained by the development of a natural defense system resulting in the synthesis of a multitude of antimicrobial molecules, which enable them to fight effectively against the pathogenic microbes [8, 9].

Anthraquinones are divided into two types: alizarin and emodine [10]. The alizarin type is used as natural dye in the textile industry [11], while the emodine type was formerly used as a like laxative compound [12–14]. Many studies have reported on antimicrobial activity of anthraquinone compounds of the emodine type [2, 15, 16]. Their variety and the continuuos discovery of new emodine derivate molecules always call in question, the specific properties of the most antimicrobial effective compounds. Moreover, the number of molecules extracted from the biological and/or potentially existing systems is by far higher than the capacity of analysis of their biological properties.

Facing these limitations, a solution consists of building models which allows for correlating the activity to structure within a family of compounds, hereby increasing the effectiveness of high throughput screening [17]. On the basis of their physical and chemical properties, the antimicrobial activity of natural substances can be predicted in order to have information on biomechanism, gain time of bio-prospection of new molecules and to study their use in the sectors of the production of antiseptics, disinfectants and drugs [18, 19]. The structure activity relationship (SAR) or the quantitative structure activity relationship (QSAR) offers approaches which could be useful to predict these antimicrobial activities according to the physical and chemical properties of the molecules concerned. This approach could constitute a first stage of molecules screening and thus making it possible to reduce the number of compounds to be tested in the laboratory.

The purpose of this study was hence to establish a relationship between antimicrobial activities and physicochemical properties of some anthraquinone molecules of emodine type isolated from *Vismia laurentii*.

## Methods

### Plant material and purification

The roots and leaves of *Vismia laurentii* De Wild were collected in March 2004 in Mbalmayo, located in the Center Region of the Republic of Cameroon and identified by Mr Nana (plant taxonomist) of the *National Herbarium* of *Cameroon*, Yaounde. A voucher specimen (N° 1882/SRFK) documenting the collection was deposited.

The extraction and purification were carried out according to [1, 2]. Briefly, air dried powder of the roots of *Vismia laurentii* (2 kg) was extracted exhaustively at room temperature with methanol (8 L) for 48 h by maceration. The suspension was filtered and the filtrate was concentrated on reduced pressure to give 100 g of brown residue. This residue was subjected to flash chromatography on silica gel (Merck, 230–400 mesh), eluted with the gradient polarity of cyclohexane and ethyl acetate to give 5 fractions labelled : A (20 g; cyclohexane), B (35 g; cyclohexane/ethyl acetate 4:1), C (18 g; cyclohexane/ethyl acetate 1:1) and D (10 g ethyl acetate). Fraction B, which according to the works of [1, 2] could contain most of the emodine type compounds based on the solvent polarity used, was further subjected to column chromatography on silica gel (Merck 70–230 mesh) and eluted with cyclohexane/ethyl acetate mixture of increasing polarity. One hundred fractions of 100 mL each were collected and analysed by TLC using the mixture of cyclohexane/dichloromethane (7:3) as mobile phase. Fractions 1–25, eluted with cyclohexane afforded three compounds which were identified as: 3-geranyloxyemodine (300 mg); compound A, friedelin (25 mg) and stigmasterol (35 mg). Fractions 27–47, eluted with the mixture of cyclohexane/ethyl acetate (9:1) gave 1.3 g of brown residue which was subjected to further column chromatography to yield laurentixanthone (25 mg), 3-methoxyemodine (25 mg); compound C and compound E bivismiaquinone (40 mg). Fraction A eluted with cyclohexane/ethyl acetate (4:1), gave after repetitive column chromatography, kampherol (16 mg), laurentixanthone A (50 mg), 1,7- dihydroxy xanthone (18 mg), vismiaquinone B (50 mg); compound B,, 2-isoprenyl-3-methoxyemodine (22 mg); compound D. The chemical structure of each isolated compound was established on the basis of their NMR spectra (one and two dimensions) [2, 20] and data recorded on BRUKER DRX-400 instrument.

### Physicochemical properties determination

The Compound polarity (Rf) was assessed by Thin Layer Chromatography method [21, 22]. The number of hydrogen bond acceptors (HA) and donors (HD) were assessed by calculations with available equations [1, 23]. Partition coefficient (LogK_O/W_), water solubility (S_W_), superficial tension (S_tens°) and polarizability (Polarz) properties were obtained by using the following predicting softwares: SMILES Translator and Structure File Generator, ACDLABS and EPIWEB version 4.1.

### Data set

**Fig. 1:**
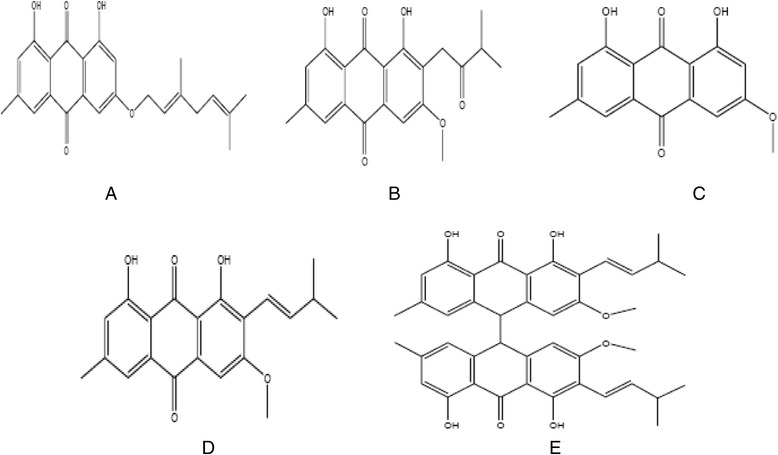
Chemical structures of chemical compounds used in this work. **a** 3-geranyloxyemodine, **b** Vismiaquinone B, **c** 3-methoxyemodine, **d** 2-isoprenyl-3-methoxyemodine, **e** Bivismiaquinone

Six (06) microorganisms consisting of three Gram positive (*Bacillus cereus* ATCC 11966, *Listeria monocytogenes* 56 Lγ and *Staphylococcus aureus* NCTC 10652), two Gram negative (*Escherichia coli* 555, *Salmonella enteritidis* 155A) and one yeast of the species *Candida albicans* were tested for their sensitivity to 5 emodine derived compounds: 3-geranyloxyemodine, vismiaquinone B, 3-methoxyemodine, 2-isoprenyl-3-methoxyemodine and bivismiaquinone (Fig. 1). Microorganisms were obtained from copies stored at −80 °C and subcultured twice in Brain Heart Infusion broth at 37 °C for bacteria and 25 °C for yeast.

### Growth kinetic of microorganisms and antimicrobial activity of selected compounds

The microbial counting was performed by dilution and seeding method on Mueller Hinton agar medium (Oxoid, Basingstoke, UK) for bacteria [24] and microscope direct counting for yeast [25] using Mueller Hinton broth medium (Oxoid, Basingstoke, UK). Antimicrobial activity was performed by macrodilution method in liquid medium for the MBC/MFC (Minimal Bactericidal Concentration/Minimal Fungicidal Concentration) according to [26].

### Statistical analysis

The Quantitative structure activity relationship was established by regression analysis using Statistica.7 of Statsoft.

## Results

### Microorganisms growth kinetics

**Fig. 2:**
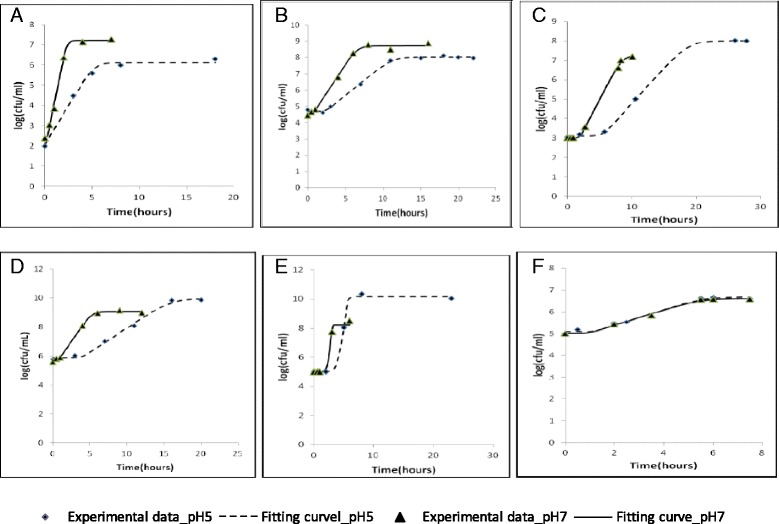
Growth kinetics of microorganisms: Gram positive bacteria: *Bacillus cereus* (**2a**), *Staphylococcus aureus* (**2b**) and *Listeria monocytogenes* (**2c**); Gram negative bacteria: *Escherichia coli* (**2d**) and *Salmonella enteritidis* (**2e**); *Candida albicans* (**2f**) in BHI medium at pH5 and pH7

In order to assess the impact of pH on the antimicrobial properties of the tested compounds, growth kinetics of the microorganisms were first obtained in those conditions and are presented for each microorganisms at pH5 and pH7 in Fig. 2. This kinetics showed that pH variation of the medium does not affect the lag and growth rate of *Candida albicans* while for the other strains, these parameters are affected. In general, it can be observed that the lag was increased and the growth rate reduced when pH was 5 compared to pH7, independently on the bacteria strain. Moreover, *Listeria monocytogenes* a Gram positive strain grew to higher final cell load notwithstanding their slow growth rates.

### Minimal bactericidal (MBC) and fungicidal (MFC) concentration

Sensitivity test reveals that reference molecules (gentamicin for bacteria and nystatin for yeast) are more active than the tested molecules which were more active on *Candida albicans*. Gram negative bacteria were less sensitive than Gram positive bacteria (Table 1).

**Table 1:** Minimal bactericidal (MBC) and fungicidal (MFC) concentration (ppm) of compounds tested against selected microorganisms

		Compounds						
		A	B	C	D	E	N	G
Yeast								
*Candida albicans*	pH 5	150	600	600	1200	550	25	
	pH 6	300	600	1200	1200	1100	50	
	pH 7	1200	1200	/	1200	1100	25	
Gram-positive bacteria								
*Listeria monocytogenes*	pH 5	600	/	/	/	/		25
	pH 6	600	/	/	/	/		6.25
	pH 7	1200	1200	/	/	/		25
*Staphylococcus aureus*	pH 5	600	1200	1200	/	1100		75
	pH 6	600	/	1200	/	/		˂ 3.12
	pH 7	1200	/	75	150	/		˂ 3.12
*Bacillus cereus*	pH 5	300	/	/	/	/		˂ 3.12
	pH 6	600	/	/	/	/		˂ 3.12
	pH 7	600	/	/	300	/		˂ 3.12
Gram-negative bacteria								
*Salmonella enteritidis*	pH 5	/	/	1200	/	/		600
	pH 6	/	/	/	/	/		600
	pH 7	/	/	/	/	/		˂ 3.12
*Escherichia coli*	pH 5	/	/	1200	/	/		12.50
	pH 6	/	/	/	/	/		6.25
	pH 7	/	/	/	/	/		25

*A* = 3-geranyloxyemodine, *B* = vismiaquinone B, *C* = 3-methoxyemodine, *D* = 2-isoprenyl-3-methoxyemodine, *E* = bivismiaquinone, *G* = gentamicin (antibacterial), *N* = nystatin (antifungicidal), <= inferior,/= not active at concentration ≤ 1200 ppm

In general, the sensitivity of the strains to all the compounds tested decreased with increase in pH. As exception to this rule, compounds C and D were more active at pH7 than at pH5 and 6. While almost all the compounds were active on *Candida albicans,* this was not the case for bacteria strains.

### Compounds physicochemical properties

Bivismiaquinone (E) had the highest partition coefficient while 3-methoxyemodine (C) had the lowest. These results were confirmed by the water solubility property of the compounds. In fact, compound C had the highest water solubility coefficient. Regarding the superficial tension, which is the tendency of a compound to contract due to internal forces and resist external forces, it was noticed that 3-geranyloxyemodine (A) had the lowest superficial tension (52) while 3-methoxyemodine had the highest (63.7). Compound C also had the lowest polarizability while compound E had the highest. Regarding the electron bond donors (HD) and electron bond acceptors (HA), bivismiaquinones (E) proved to be quite different from the other compounds by demonstrating an HD of 4 and HA of 8. This was also the case for the insaturation number (IN) where compound E was far more unsaturated than the other compounds. The polarity degree also confirmed the Log K_o/w_ and Sw results, indicating that compound C was the most polar (Table 2).

**Table 2:** Physicochemical properties of compounds tested in this work

QSAR indipendent variables^a^	Tested compounds				
	A	B	C	D	E
Log K_O/W_	8.67	4.950	4.570	6.790	13.450
S_W_ (mg/L)	3.25^a^10^−5^	0.083	0.563	0.003	4.93^a^10^−11^
S_tens°	52	58.600	63.700	57.800	59.300
Polarz	45.760	38.600	29.310	39.260	77.650
MW (g/mol)	406.17	362.00	284.06	352.00	674.28
HD	2	2	2	2	4
HA	5	6	5	5	8
R_f_	0.80	0.76	0.86	0.80	0.60
IN	13	12	11	12	23

*Log K*_*O/W*_ partition coefficient, *S*_*W*_ water solubility, *S_tens* superficial tension, *Polarz* polarizability, *MW* molecular weight, *HD* hydrogen bond donor, *HA* hydrogen bond acceptor, *R*_*f*_ polarity degree, *IN* insaturation number

^a^The Compound polarity (Rf) was assessed by Thin Layer Chromatography method. The number of hydrogen bond acceptors (HA) and donors (HD) were assessed by calculations with literature available equations. Partition coefficient (LogK_O/W_), water solubility (S_W_), superficial tension (S_tens°) and polarizability (Polarz) properties were obtained by using the following predicting softwares: SMILES Translator and Structure File Generator, ACDLABS and EPIWEB version 4.1

### Structure-activity relationship

Quantitative relationship between structure and activity of molecules (QSAR) was possible only for data obtained with *Candida albicans*. In other to perform this, the MIC (Minimum Inhibitory Concentration) at pH7 for compound C was assessed by increasing the maximum concentration limit tested and found to be 3000 ppm. First, all the data for *Candida albicans* were merged together irrespective of the pH and a regression analysis was performed in order to obtain a quadratic polynomial equation. Unfortunately, no significant result was obtained. After hypothesizing that the data set could not be sufficient to explain the effect of pH on the CMF, we decided to split the regression analysis of each pH. Equations 1, 2 and 3 report the polynomial equation indicating the relationship between statistically significant physicochemical parameters and the antifungal potential (Log MFC) of the anthraquinones studied. QSAR Models obtained for the activity of tested molecules on *Candida albicans* can thus be written in the following equations where the increase of Log MFC indicates a reduction of the antifungal activity of the compound.

1 $$ \mathbf{Log}\ \left(\mathbf{MFC}\right)\ \mathbf{at}\ \mathbf{p}\mathbf{H}\mathbf{7} = \mathbf{1.671} + \mathbf{0.488}*\mathbf{Log}{\mathbf{K}}_{\mathbf{o}/\mathbf{w}} + \mathbf{0.774}\ *\mathbf{H}\mathbf{A}\ \hbox{--}\ \mathbf{0.147}\ *\mathbf{Polarz} $$

2 $$ \mathbf{Log}\ \left(\mathbf{MFC}\right)\ \mathbf{at}\ \mathbf{p}\mathbf{H}\mathbf{6} = \mathbf{2.410}\ \hbox{--}\ \mathbf{0.077}*\mathbf{M}\mathbf{W} + \mathbf{1.321}*\mathbf{H}\mathbf{A} + \mathbf{0.537}*\mathbf{Polarz} $$

3 $$ \mathbf{Log}\ \left(\mathbf{MFC}\right)\ \mathbf{at}\ \mathbf{p}\mathbf{H}\mathbf{5} = \mathbf{2.197}\ \hbox{--}\ \mathbf{0.092}*\mathbf{M}\mathbf{W} + \mathbf{1.620}*\mathbf{H}\mathbf{A} + \mathbf{0.642}*\mathbf{Polarz} $$

At the different pH, the common independent variables that significantly affected the Log (MFC) were the polarizability (tendency of a compound to negatively charge itself and hence be easily modified in the presence of positively charged compounds) and the number of hydrogen acceptors (HA). As the pH of the inactivation medium decreased, the effect of those two variables on the fungicidal properties increased as it can be observed from the coefficients. At pH7, the octanol/water partition (Log K_o/w_) which describe the affinity of a compound to lipid or water phases, had a significative impact on the fungicidal property while at pH6 and 5, it was the molecular weight (Table 3).

**Table 3:** Statistically significant parameters obtained by multiple regression analysis, and by correlations between experimental and calculated values

Parameters	pH 7				pH 6				pH 5			
	coef ± errSt	IC_f_ 95%	IC_s_95%	P	coef ± errSt	IC_f_ 95%	IC_s_95%	P	coef ± errSt	IC_f_ 95%	IC_s_95%	P
Const	1.671 ± 0.072	1.531	1.813	0	2.410 ± 0.156	2.104	2.716	0	2.197 ± 0.206	1.792	2.601	0
Log K_o/w_	0.488 ± 0.020	0.448	0.528	0	NS	-	-	-	NS	-	-	-
MW (mol/g)	NS	-	-	-	−0.077 ± 0.009	−0.094	−0.059	0	0.092 ± 0.012	−0.116	−0.069	0
HA	0.774 ± 0.031	0.714	0.835	0	1.321 ± 0.142	1.043	1.598	0	1.620 ± 0.187	1.254	1.987	0
Polarz	−0.147 ± 0.006	−0.158	−0.135	0	0.537 ± 0.065	0.409	0.665	0	0.642 ± 0.086	0.473	0.810	0
R^2^ Model		0.995				0.943				0.936		
SSE Model		0.001				0.015				0.026		

*coef ± errSt* parameters coefficient, *P* probability of kindness of the equation, *Const* constancy, *MW* molecular weight, *Log K*_*o/w*_ *(or Log P)* partition coefficient, *Polarz* polarizability, *HA* hydrogen bond acceptor, *NS* non significative, *IC*_*f*_ *95% and IC*_*s*_ *95%* inferior (IC_f_) and superior (IC_s_) confidence interval, correlation values: R^2^ model and SSE model

The quantitative structure activity relationship (QSAR) on bacteria could not be performed because of the low activity of the compounds. However, it was possible to propose a structure activity relationship (SAR). In fact, by observing the results, it can be noticed that 3-geranyloxyemodine (A) was bactericidal to the three Gram positive bacteria strains with activity increasing with decreasing pH while 3-methoxyemodine (C) was active only on *Staphylococcus aureus* with activity decreasing with pH. On the other hand 2-isoprenyl-3-methoxyemodine (D) was active only at pH7 and only on *Staphylococcus aureus* and *Bacillus cereus*. In terms of decreasing antimicrobial activity, the tested compounds can be classified in the following way: 3-geranyloxyemodine (A) > 3-methoxyemodine (C) > 2-isoprenyl-3-methoxyemodine (D) > vismiaquinone (B) > bisvismiaquinone (E). It can be noticed that compound E is an association of two molecules of compound D by the presence of a ketone group on the isoprenyl substitution in position 2. This substitution in position 2 is the difference between compound C and compounds B and D. Finally, compound A differs from compound C by the length of the aliphatic chain of the methoxy substitution. It can hence be assumed that steric effect, weight and the presence of substitutions in position 2 of emodine derivatives is detrimental to their bactericidal activity while increase in the aliphatic chain length of the methoxy substitution in position 6 is beneficial to the antibacterial activity of these emodine derived anthraquinones.

## Discussion

The differences observed during the growth kinetics at pH5 and 7 of the tested strains can be associated to the different nature of their cell walls. In fact, the effect of pH that is mostly described on the cell internal pH [27] is most important on bacteria than on fungi. Moreover, homeostasis regulations that sometimes involve ATP dependent processes may also include cell membrane lipid modifications to reduce fluidity [28]. Fungi resistance to pH also depends on their high cell wall thickness and composition mainly made of 80–90% glucomannoproteines, glucanes and chitins; this last compound being higher in *Candida albicans* with respect to other yeast species [29].

The emodine derived compounds tested in this work were highly colored and hence permitted only evaluation of the MBC and MFC. The microbiocidal concentrations observed for the different compounds can be explained by the compound interference with the cell wall, the membrane, nucleic acid and enzymes [30, 31]. The presence of an external membrane on the Gram negative bacteria can explain the difference of sensibility observed between the two groups of bacteria. Kosanić and Ranković [32] suggested that the cell wall structure and composition of bacteria and fungi could account for the different sensitivity to antimicrobial compounds. On the other hand, Gram positive bacteria and *Candida albicans* cells have their cell walls exposed, and compounds that can interact with these cell walls should have a long aliphatic chain to help disorder the cell wall. This is the case of compound A compared to compounds C and D. Sikkema et al. [33] observed that saturated alcanes had very low antimicrobial activity, while [34] have demonstrated that unsaturated aldehydes had more antimicrobial activity than saturated ones. This explains the difference between compounds D and B. Moreover, another possible mechanism was proposed by [35] who demonstrated that the antimicrobial activity of emodine on *Helicobacter pylori* was also due to the interference with saturated and unsaturated fatty acid elongation by inhibiting the β-hydroxyacyl-Acp dehydratase (HpFabZ).

Regarding the physicochemical properties and their relation with the antimicrobial activity of the compounds tested, different methods as described before were used to assess them. The molecular weight (MW) which is an indication of a compound’s steric effect is also one of the key parameters of compounds with pharmacological properties (Lipinski et al., [23]). This author also proposes the ideal molecular weight to be lower than 500 g. The numbers of electron donors (HD) and acceptors (HA) indicate the capacity of a compound to form hydrogen bonds with the cell membrane compounds. Moreover, the partition coefficient (Log K_o/w_) gives information on the lipophilicity of the compound and hence its capacity to distribute itself in the membrane lipidic phase and the aqueous environmental or cytoplasmic phase. Regarding the degree of polarization, it is the tendency of a compound to negatively charge itself and hence be easily modified in the presence of positively charged compounds. The surface tension on the other hand represents the internal attractive forces of a compound that limits bond creation with other compounds. The QSAR obtained for *Candida albicans* indicated that HA and Polarz were the common significant variables of the equations for each pH. Both physicochemical parameters are associated to the compound capacity to bind to other molecules by forming bonds with positively charged atoms. In fact the growing importance of HA and Polarz in the equations as pH decreases (protons concentration increase) in the environment confirm this. At pH7, the concentration of protons is lower and hence the increase of the Polarz negatively affects the fungicidal property. The fact that the antifungal property of the compounds depended on the pH, and that the pH did not affect the growth of *Candida albicans* may suggest that the effect of pH on the compounds antimicrobial effect is associated with compound modification.

The logical structure activity relationship deduced for the bacteria strains tested indicates that the substitution in position 2 of the emodine compound is detrimental for the antibacterial activity of these compounds while the insaturation of the substitute isimportant for this activity. Moreover the increase of the aliphatic chain length of the methoxy substitute in position 3 increases the lipophilicity of the compound. The antimicrobial property which is increased by the lipophilicity of the compound is reduced as the compound molecular weight increases. In fact the lipophilicity associated to the Log (Ko/w) denotes the capacity to integrate the cell wall and membrane capacity which may be slowed by the steric effect of the compound, revealed here by the molecular weight.

## Conclusion

In conclusion, the present work has demonstrated the antifungal and antibacterial properties of some anthraquinones of emodine type isolated from *Vismia laurentii*. This antimicrobial property is increased by the presence of a long aliphatic chain methoxy group substituted in position 2 of the emodine structure. The mathematical equations produced demonstrate that QSAR can contributeto understanding the diversity of compound antimicrobial activity present in plant extracts.

## Abbreviations

coef ± errSt: Parameters Coefficient
Const: Constancy
G: Gentamicin
HA: Hydrogen bond acceptor
HD: Hydrogen bond donor
IC_f_ 95%: inferior Confidence Interval
IC_s_ 95%: superior Confidence Interval
IN: Insaturation Number
Log K_O/W_(or Log P): Partition coefficient
MBC: Minimum Bactericidal Concentration
MFC: Minimum Fungicidal Concentration
MIC: Minimum Inhibitory Concentration
MW: Molecular weight
MW: Molecular weight
N: Nystatin
NS: Non significant
P: Probability of kindness of the equation
Polarz: Polarizability
QSAR: Quantitative Structure Activity Relationship
R_f_: Polarity degree
S_tens°: Superficial tension
SAR: Structure Activity Relationship
S_W_: Water solubility
